# Book Reviews

**Published:** 1801-05

**Authors:** 


[ To be continued. ]

### CRITICAL RETROSPECT

### OF

### MEDICAL AND PHYSICAL LITERATURE.

### [ FOREIGN AND DOMESTIC. ]

*Anleitung zur Kentniss und Wahl des Arztes*; i. e. Inſtruction for the Knowledge and Choice of a Phyſician. By JOSEPH FRANK, Phyſician to the General Hoſpital at Vienna. 123 pp. 8vo. 1800. Vienna, Schaumburg.

THIS publication is intended to inform the public of the qualities of a good phyſician, in order to enable it to judge properly, whether the phyſician, to whom health and life are to be entruſted, really poſſeſſes all the knowledge and abilities which are neceſſary for the preſervation of health and the cure of diſeaſes. It is very obvious from daily experience, that with reſpect to the choice of a phyſician, the public is frequently miſled by faſhion, deceived by an ambiguous celebrity, by recommendations, and by protections; and we are afraid that this will by no means be remedied by the preſent publication. For though we do not deny to it the merit of acquainting the public with the true qualities of a phyfician, by which it may be enabled to judge in fome fingle inftances, yet on the whole, it would require a greater knowledge of the medical fcience than can be expected, but without which, however, it will be impoffible to difcover any miftakes or errors that a phyfician may commit in the treatment of a difeafe. After having premifed fome juft remarks on the too great number of thofe who devote themfelves to medicine, and on the different means and expedients by which yonng doctors attempt to introduce themfelves into practice, the author treats, in the *firft fection,* of the perfon of a phyfician. *Ch.* 1. Of the exterieur of the phyfician, to which he refers his phyfiognomy, drefs, and manners. *Ch.* 2. Of the phyfical qualities of a phyfician. Medical art being founded upon experience, and accurate obfervation of things that are obvious to our fenfes, a phyfician ought to poffefs them in a moft perfect ftate; this the author fhows of every fenfe in particular. *Ch.* 3. Of the moral qualities, judgment, &c. of a phyfician. Humanity, patience, modefty, fecrecy, and moderation, are the principal virtues at which a phyfician fhould particularly aim; befides a comprehenfive genius, he ought to have a good memory, and a knowledge of the dead and living languages. The *fecond fection* treats in *three chapters,* of the conduct of the phyfician towards his colleagues, towards furgeons and apothecaries, and towards the patient; upon the laft of which he particularly dwells; and we meet every where with remarks that are confirmed by experience. At the end of the preface the author promifes to prefent to his readers a fketch of the Brunonian fyftem, a circumftance which fhould not furprife us, as the author is known to be one of the principal partizans of the Brunonian theory, and confequently interefted in its acquiring the moft extenfive popularity.

---

*Recherches Phyfiologiques fur la Vie et la Mort;* i. e. Phyfiological Refearches on Life and Death; by XAV. BICHAT, Profeffor of Anatomy and Phyfiology. Paris, Year VIII. in two vol. 8vo. price 5 francs, or about 5s.

WE here announce a new work on two interefting fubjects of the organic doctrine, *life* and *death,* which, on account of its general excellency, deferves a more copious notice. The author attempts to enquire into the Zoonomick laws by analyfis and ftrict induction, and has, indeed, given light on feveral obfcure doctrines of phyfiology, by his vivifections, and other ingenious experiments; though, on the other fide, he has frequently fuffered himfelf to be deluded by a luxuriant imagination, and to be mifled by an acutenefs more fubtle than true, with which he endeavours to force Nature into fyftematical bonds, which are frequently difregarded in a fubject that will never allow itfelf to be conftrained by fyftems.

The work is divided into two parts, the firft of which treats of Life, and the fecond of Death. The firft part comprehends ten articles, articles, viz. Art. 1. *General division of life.* The author calls life the whole (l'enfemble) of the functions that refift death. The principle of life, which we only know from its phenomena, oppofes itfelf to the deftructive action of external influences; and there exifts, confequently, a continual viciffitude of action and re-action in the living body. *Life* fhows two principal modifications, an *organic* or *internal* life, and an *animal* or *external* one, *(vie animale, ou de relation)* the firft of which is common both to animals and vegetables, comprehending their exiftence, nutrition, growth, affimilation, digeftion, fecretions, and excretions; the fecond belongs to men and animals, by which they are enabled to perceive, to move voluntarily, and to exprefs their defires and paffions; in one word, to connect their exiftence with other beings. Each of thefe two kinds of life has again two orders of functions. The firft order of the animal life proceeds from without, towards the brain; the fecond, from the brain towards the organs of locomotion and of voice. By the firft order of functions in the organic life, the nutritive fubftances are affimilated, to which belong digeftion, circulation, refpiration, and nutrition: By the functions of the fecond order, the particles that have become heterogeneous to organization are removed; abforption, circulation, perfpiration, and fecretion. The fyftem of circulation, as the centre of the organic life, belongs to both orders.

Art. 2. *Chief differences of the two lives, with regard to the forms of their refpective organs.* The greateft fymmetry prevails in the organs of animal life; whereas thofe of the organic life are extremely irregular. By a line drawn along the middle of the human body, the organs of the firft life, brain and nerves, will be feparated in two equal parts, which are not ftrictly dependent on each other, as we fee, for inftance, in hemiplegia; but in the organic life, every part is combined and fubfervient to each other; their form and fituation are, at the fame time, more frequently changed than in the other. The idea of this law, the author firft difplayed, with a great deal of acutenefs and ingenuity, in the Memoirs of the Medical Society at Paris; however, it is liable to many objections that may be juftly raifed againft it.

Art. 3. *Chief difference of the two lives, with regard to the mode of action of their refpective organs.* The actions of the organs of animal life proceed with the greateft proportion and harmony, which feems in a great meafure to be owing to their fymmetrical fituation and form. The author, in ftrictly examining this law, meets with feveral exceptions, which, however, he endeavours to explain with much acutenefs. The apparent inequality between the actions of the mufcular fyftem of the right fide, which is thought to enjoy more ftrength than that of the left, is not founded in nature, but merely produced by the cuftoms of fociety. In the mode of actions of the organic life, however, the greateft difference prevails, as one organ is frequently larger than another, and performs its functions better than another; one kidney is often larger than the other, and fecretes more urine, &c.

Art.

Art. 4. *Chief difference of the two lives, with regard to the duration of their actions.* The functions of the organic life proceed in a continual progreffion, whereas in the animal life, an intermiffion takes place: The author attempts to elude feveral objections that may be propofed againft this law, which does not at all appear to be founded upon obfervation. It cannot be well afferted, that all influence of the nerves, upon voluntary mufcles, ceafes when they are in a quiet ftate, or on the organs of fenfes during fleep.

Art. 5. *Chief difference of both lives, with regard to cuftom and habit.* Cuftom diminifhes the perception and fenfe of pain and pleafure, but it brings the judgment to a greater perfection: The power of habit does not extend to the organic life, and the circulation, refpiration, and perfpiration, are not influenced by it. The author, however, does not omit to remember the great power of habit in eating and drinking, return of appetite, excretio alvi et urinae, on which account he refers them to the phenomena that keep the medium in both lives.

Art. 6. *Chief difference of both lives, with regard to moral properties.* All operations of the underftanding are fituated within the compafs of animal life, but the paffions are ruled by the organic life, whofe found and morbid ftate, different degree of ftrength, and the predominant functions of any organ, have a remarkable influence on paffions and temper. The influence of violent paffions on the mufcular fyftem, ought to be explained, according to our author, from the more or lefs vehement influx of blood into the brain, and the locomotive organs, &c. But as the debilitating and depreffing affections of the mind do always leave vifible impreffions in the voluntary mufcles, particularly of the face, and as, befides, lefions of the inner organs produce convulfions and palfies of the extremities, the author is obliged to adopt a fympathetic connexion between the organs of the internal life and the brain, in order to explain thofe phenomena. He denies that the paffions arife from the *centrum epigaftricum* of nerves, an opinion which fome modern phyfiologifts have adopted: He adds, however, fome very ingenious remarks on the great fympathetic nerve, in which he difplays at the fame time a profound knowledge of of the finer anatomy.

Art. 7. *Chief difference of the two lives, with regard to the vital powers.* The vital powers are not fubject to phyfical laws; extenfibility, a property of the tela cellulofa, which even remains after death, differs greatly from fenfibility and contractility. The firft, however, is of a different character in the two lives, becaufe in the organic life it confifts in the faculty of receiving impreffions, but in the animal life of conducting them to a common central point; and though this animal and organic fenfibility agree in their chief character, yet they feem to be diftinguifhed by the degree and proportion with which they act. Contractility may be feparated into animal and organic; the firft of which is influenced by the will, and begins in the fenforium commune; the fecond exifts in the different organs, and admits two modifications, the perceptible and imperceptible contractility.

Art.

Art. 8. *Origin and progress of the animal life.* The activity of the organic life begins in the firſt moment of exiſtence, but in the embryo animal life cannot be traced. The actions of the animal organs come to perfection by degrees, whereas the organic ones perform their functions from the firſt moment to the end with equal perfection and accuracy. The new-born animal ſtaggers in walking and ſtanding, while its ſtomach digeſts well, and the glandular ſyſtem acts in juſt proportion.

Art. 9. *Origin and progress of the organic life.* The functions for aſſimilation are very ſimple in the fetus, and proceed very quickly, as the nutritive particles enter into the fetus after being prepared in the mother. The ſecretions and excretions are ſlow and inconſiderable; but as the inner organs receive immediately the juſt degree of action, none of them ſhould properly excel the other, or become ſtronger or weaker. This, however, is not found to be ſo; the liver, for inſtance, frequently rules other organs, whence the varieties in the temper.

Art. 10. *Natural death of both lives.* The ſame difference which they ſhow in their beginning and progreſs, are alſo remarked in their ending. Animal life ceaſes in natural death a long time before the organic. The taſte remains the longeſt. Mankind approaches, in the boſom of the mother, and at the verge of life, to a plant. Natural death has two periods, in the firſt of which reſpiration and circulation ceaſe together with the animal life; in the ſecond, the reſt of the organic functions, but by degrees and ſlowly. The digeſtive power of the ſtomach and abſorption continue for ſome time; the hair and nails grow, &c. The death of old age proceeds in a different manner; one function is extinguiſhed after another; circulation is ſtopped firſt in the ſmaller, then in the larger veſſels; in ſhort, the death of old people proceeds from the periphery towards the centre, and the heart is the *ultimum moriens.*

[ To be continued. ]

---

*Observations on Mr. Home's Treatment of Strictures in the Urethra; with an improved Method of treating certain Cases of those Diseases,* by Thomas Whately, Member of the Royal College of Surgeons in London. Illuſtrated by an Engraving, 8vo. pp. 112, price 2s. 6d. London, 1801, Johnſon, &c.

We are much pleaſed to find that the hope we expreſſed in our account of Mr. W's correct and elaborate work on Gonorrhœa Virulenta, (vol. v. p. 383) has been already ſo far gratified. An improper treatment of gonorrhœa is, doubtleſs, the moſt frequent cauſe of ſtrictures, and therefore we may conſider the treatment of ſtrictures in the urethra as forming alſo an important part of the cure of the venereal diſeaſe, or its conſequences. The male urethra has, with ſufficient reaſon, been called Pandora's box; and of all the evils it has produced, ſtrictures, either immediately or in their conſequences, may be conſidered as the moſt formidable and diſtreſſing. However "invidious," therefore, "it may appear to others, others, or however painful to ourfelves," the author obferves, "it may be, to attack the opinions of a refpectable character; yet to be more influenced by a reluctance to condemn the practice of an individual than by a defire to relieve the thoufands of wretched beings around us, would be a reprehenfible delicacy." Our narrow limits do feldom permit us to do juftice to practical works of this kind; and indeed our readers muft long have obferved, that our object is merely to point out and diftinguifh fuch new works as merit the attention of certain practitioners, or the public in general. Now it is our opinion, that no furgeon who wifhes to do his duty to his patients in the beft manner in his power, will neglect to read Mr. W's pamphlets on Gonorrhœa and Strictures: That our readers, however, may have an opportunity of judging for themfelves, rather than relying on our authority only, we fhall, according to our cuftom, prefent them with a few extracts. After making fome remarks on the predifpofing caufe of ftrictures in the urethra, Mr. W. proceeds "to inquire into the prefent method of treating certain cafes of them by the cauftic. My obfervations on this head are meant, fays he, to excite the attention of the reader to an improved method of treating the complaint in queftion. Fearing, however, left fo many publications on the cure of ftrictures of the urethra by the cauftic fhould miflead the young practitioner, and caufe him to overlook what has been done, and what ftill may be done by a judicious management of the common bougie, I have judged it right, before I enter on the fubject of cauftic applications, to make a few extracts refpecting the ufe of this fimple inftrument, from thofe excellent furgeons, Mr. S. Sharpe and Mr. Hunter.

"Strictures in the urethra appear not to have been treated judicioufly, previous to the time of Monfieur Daran. Although the bougie had been ufed long before his time, it is to him we are indebted for a more extenfive and methodical application of it. Mr. Samuel Sharpe, and Mr. Hunter, have fince extended and brought to great perfection the ufe of this inftrument, by which a very diftreffing complaint has been, in many inftances, perfectly cured; and in the greater number of other cafes, it has been fo much alleviated by its occafional ufe, as to enable the patient to pafs through life without fuffering any ferious inconveniences from his infirmity. Both Mr. Sharpe and Mr. Hunter (particularly the latter) have given very excellent directions how to ufe this remedy, and have informed us upon what principle it acts."

Mr. W. concludes his quotations from thefe authors, with the following from Mr. Hunter:

"And in the following fection he remarks, that "When a bou-
"gie can readily pafs, there is no neceffity for ufing any other
"method to remove the ftricture." He then proceeds with great judgment to the treatment by cauftic of fuch ftrictures, "as are
"either fo tight as not to allow the fmalleft bougie to pafs;" or of fuch, "as have not the orifice in the ftricture in a line with the
"urethra, which will make it uncertain, if not impoffible, to
"pafs a bougie." He likewife recommends the cauftic in thofe cafes cafes, in which "there may be no paffage at all, it having been "obliterated by difeafe, and the urine paffed by fiftulæ in perinæo."

"Mr. Hunter applied the cauftic in cafes of this defcription at firft by means of a filver canula; but, on account of many difadvantages attending this mode of ufing it, we are told by Mr. Home, that, for fome years before Mr. Hunter's death, he ufed it upon an improved plan, viz. by inferting a fmall piece of lunar cauftic into the end of a bougie, in fuch a manner, that the cauftic was furrounded every where laterally by the fubftance of the bougie; fo that in its paffage to the ftricture, the cauftic was fcarcely allowed to come into contact with any part of the membrane of the urethra; the point of the bougie always moving in the middle line of the canal.

"Mr. Home informs us, that, ever fince Mr. Hunter's death, he has continued to make ufe of the fame method of applying the cauftic; but it is evident from many parts of his work, that he employs commonly, for this purpofe, bougies of a much larger fize than thofe which were ufed by Mr. Hunter; and he feems to be defirous, in every cafe in which he adopts this method of treatment, to pafs through the ftricture a bougie, thus armed with cauftic, as large as the natural fize of the canal of the urethra.

"Mr. Home has not only employed the cauftic in thofe cafes, in which it had been recommended by Mr. Hunter, but he has likewife ufed it in old ftrictures, and fuch as could not be dilated to more than half the fize of the reft of the canal by the common bougie, and which, when dilated, were apt to contract, and require a frefh application of the bougie within two months after it had been left off. Mr. Home therefore informs us, that his obfervations "will be confined entirely to the treatment of thofe ftrictures which admit a fmall bougie to pafs into the bladder; and "that they are publifhed with a view to extend the ufe of the "cauftic to a greater variety of cafes, and in fome meafure upon "a different principle from that on which it was applied to impervious ftrictures by the late Mr. Hunter."

"The fubject is of great importance to thoufands afflicted with this malady. And as the propofed alteration in the plan of cure is by a remedy of a very violent nature, capable, if not ufed with great judgment, of producing confequences worfe than even the difeafe for which it is applied, it demands the moft ferious inveftigation. This I fhall now enter upon, by firft making a few obfervations, concerning the manner in which ftrictures ufually affect the urethra, the number they commonly confift of in the fame perfon, and the part of the canal in which they are ufually fituate."

Our Author has not contented himfelf with common experience or obfervation, but has made a number of experiments with cauftic on vifible parts of the body, fuch as the infide of the cheek, which refemble the urethra in ftructure. He then fays,

"Having endeavoured to fhew what are the probable effects of lunar cauftic, when applied to the inner membrane of the urethra in cafes of ftricture, by comparing its known effects when applied to parts that are open to obfervation, I fhall proceed to point out the defects which appear in Mr. Home's method of ufing it, and the confequences which fometimes refult from its being thus applied. After this I fhall, with all deference, propofe another mode of applying the cauftic, which will, I apprehend, preclude every difagreeable effect that has hitherto attended the ufe of this powerful remedy.

"I begin with obferving, that the orifice of the urethra is fo fmall in fome cafes of ftricture, as not to admit the fmalleft bougie I have ever feen armed with cauftic. Of courfe, the cauftic cannot be applied to the ftricture, while it is in this ftate. This contraction may arife, either from a natural conformation of the part, or from its having been the feat of chancres; or it may have come on gradually, from the long continuance of a ftricture in any part of the urethra. If the contraction arife from the firft caufe, it may often be remedied by dividing the web at the orifice of the urethra; but this muft render the part for fome time very tender, and in an unfit ftate to be ftretched by a large bougie. A contraction from the fecond caufe may frequently be dilated; though it muft be confeffed, that, in fome of thefe cafes, it is impoffible to dilate it, fo far as to admit even the fmalleft fized armed bougie for the purpofe of applying the cauftic. Of this I have lately had an inftance. In the third cafe, dilatation is generally practicable, and it will remove the difficulty."

The objections to Mr. Home's manner of applying the cauftic, turn on fome of the following circumftances, viz.

The mucus of the urethra renders its action uncertain.

The bougies are too large, and therefore only reach the anterior part of the ftricture, or do injury if forced through it.

The cauftic is applied too often, and to too many ftrictures at a time.

The confequences have often been ferious and alarming.

Mr. W. next proceeds to deliver his own *Method of treating Strictures in the Urethra by the Caustic;* but for this we muft refer our Readers to a future Number.

---

*A Compendious Medical Dictionary, containing an Explanation of the Terms in Anatomy, Physiology, Surgery, Practice of Physic, Materia Medica, Chemistry, &c.* By R. Hooper, M.D. 2d edit. 8vo. pr. 7s. in boards. London, 1801, Murray and Highley.

We can with great propriety recommend this Medical Dictionary, as containing a correct explanation of the terms, with their derivations, and marks to direct their proper pronunciation.

---

*Reports on the Diseases in London, particularly during the Years,* 1796, 97, 98, 99, *and* 1800. By R. Willan, M.D. F.A.S. large 12mo. pp. 360. London, 1801, Phillips, &c.

To practitioners near the metropolis this work muft be highly interefting; and as the difeafes of the poor in all large cities are very fimilar, the Hofpital and Difpenfary practititioner in every part of of the world will find much ufeful and important information in it. The Nofology of feveral of the cutaneous difeafes will require a reference to Dr. Willan's elaborate work on that fubject, of which we are forry to obferve, a part only is at prefent publifhed. Dr. W. gives the following account of the hiftory of the prefent volume.

" A part of the following Reports was inferted in the Monthly Magazine for the years 1796, 1797, and in the Medical and Phyfical Journal for 1799. Many of the readers of thofe periodical works having honoured with particular notice the ftatements refpecting Chronic Difeafes, as well as Epidemics, it was propofed to me that the monthly accounts of them fhould be revifed, and republifhed feparately, with any additional obfervations which might occur.

" With this propofition I have complied, finding myfelf unable through a variety of engagements, to accomplifh another object which has been urged as a defirable one;—to give, under fome proper arrangement, an accurate hiftory of the diforders prevalent in or near London, from actual obfervation, without any bias from the confideration of difeafes of other climates, and without a conftant reference to the congeries of fymptoms detailed by fyftematic writers.

" In the prefent work, not only the accounts of difeafes for 1796, 1797, and 1799, are amplified, but Reports, entirely new, with various collateral obfervations, are added for the year 1798; alfo for 1800, a year remarkable for its temperature, and for an unufual feries of complaints. Any irregularities which appear in the lifts of difeafes will, I hope, be excufed, a nice arrangement of them not having been the primary object. The generic diftinctions and varieties muft be principally referred to the Nofology of Sauvages. In forming the three claffes of Acute, Chronic, and Periodical Difeafes, the ufual plan of nofologifts has not been obferved. All the writers on this fubject, influenced, perhaps, by the opinion of Sydenham, have not only included Intermittents under the denomination of Fever, but confidered one of their paroxyfms as an epitome of Fevers in general, the different forms of which are faid to confift merely of a repetition of fuch paroxyfms more or lefs diftinctly marked with longer or fhorter intervals. Thofe, however, who take the trouble to compare minutely the fymptoms of an Ague, and of a malignant Fever from contagion, will find that the primary appearances, the courfe, and crifis of the two difeafes are as different as their exciting caufes; and that no more analogy fubfifts between them than between the Small-pox, Eryfipelas, Rheumatifm, and internal Inflammation. I have, from clofe attention, been fo much impreffed with the difference, as to think that intermittents fhould be wholly disjoined from every diforder propagated by infection, and arranged in a feparate clafs. With refpect to the claffes under which the difeafes are put down in the following pages, it may be obferved, that,

" 1. Acute Disases are attended with difturbance of the bodily bodily functions, fo violent and general, that unlefs they terminate favourably, or change their form, they muft prove fatal within a fhort compafs of time. The fymptoms characterizing an acute difeafe at its commencement, and which have little remiffion during its courfe, are, fudden and confiderable lofs of ftrength, pain in the loins, aching of the limbs, total incapacity of attention or exertion of mind, heat of the fkin, thirft, a frequent pulfe, and furred tongue. Thefe general fymptoms, differently proportioned and varioufly modified, according to the ftrength, depreffion, or irregularity of the pulfe, the ftate of the mind, and appearances of the tongue, form the fub-divifions and generic diftinctions of febrile complaints.

"2. Chronic Diseases are of long duration, and moftly confift of uneafinefs in fome part or organ, and of impediment to the performance of its functions. They are ufually attended with general debility, but not with violent diforder of the conftitution.

"3. Periodical Diseases are characterized by a return, at ftated intervals, of pain and general diforder, or fits of fhivering, followed by heat of the fkin, and perfpiration, the whole being comprifed in lefs than twenty-four hours. During the intervals, however, the patient is not in a ftate of health, but has a fallow complexion, and is affected with languor, debility, lofs of appetite, &c.

"Moft of the plans of nofology are exceptionable, as being formed on hypothetical principles, rather than a ftrict analogy between the difeafes put in the fame order. It is not my prefent view to multiply objections, nor to enlarge on this fubject; I fhall only obferve that the difeafe, to which the denomination of Synochus is hereafter applied, has fome affinity with the Synochus of the antients. This term is not employed by Dr. Cullen in its original fenfe, but to exprefs the combination of an inflammatory with a contagious Fever. The practitioners of North Britain haftily exclude from nofology, and feem to deny, the exiftence of a complaint with which their ftation does not lead them to be acquainted.

"Eryfipelas is ufually ranked among the Exanthemata, though having little affinity with the other difeafes arranged under the fame order. The form of it entitled Eryfipelas phlegmonoides, does not feem communicable by contagion: However, when the fluid contained in its veficles is inoculated into the arm, it excites a diffufe inflammation and fwelling, with a flight degree of fever. The œdematic, or gangrenous forms of Eryfipelas may be combined with malignant Fever, and thus communicated from one perfon to another. Inftances of this have occurred frequently in Hofpitals, the complicated difeafe fpreading through the whole ward. It is fingular, that the Fever likewife unites itfelf with other complaints, and is propagated by infection under a double form. I may mention, as an inftance, the combination of an ulcerated Sore-throat with malignant Fever. To this combination alone, which often occurs, occurs, and is very contagious, the title of Angina Maligna would have been moſt properly applied. Medical writers, by not diſtinguiſhing it from the Scarlatina anginoſa, have been led into obſcurity, and made a foundation for ſome needleſs controverſies. I may here alſo refer to the obſervations made on the complication of puerperal and malignant Fever, which has ſome analogy with the caſes above ſtated.

"The term Hectica is uſed in the ſignification given to it by the Greek phyſicians; among whom it was not conſidered merely as a ſecondary complaint, depending on internal ſuppuration, or any local defect, but as ariſing from a failure of ſtrength in old age, from an exhauſted ſtate of conſtitution, occaſioned by fatigue, long faſting, anxiety, or loſs of ſleep; and ſometimes as a ſequel of Cauſos or other Fevers. They remark farther, that the Hectica often appears at firſt like an Ephemera, that it is always aggravated after food, that its duration is indefinite, and that it often terminates in a maraſmus. All the ſpecies of Hectic are characterized by the recurrence every twenty-four hours, or ſometimes every twelve hours, of heat of the ſkin, after ſlight chillineſs, with a circumſcribed fluſh of the cheeks, an increaſed velocity of the pulſe, and violent perſpirations towards morning.—In infancy, childhood, youth, and old age, Hectic takes place, without any local affection, from changes in the conſtitution, connected with the different ſtages of human life. A ſimilar ſtate of diſorder is often produced in perſons of the middle age, when the conſtitutional vigour firſt appears to decline, not reſiſting as uſual the operation of cold, fatigue, and other occaſional cauſes. This ſtate is moſtly accompanied with aphthous ulcerations of the tongue and fauces, and a large ſecretion of frothy phlegm. Under this head alſo muſt be ranked the Febris aphthoſa, or Hectica aphthoſa, often put down in the ſucceeding liſts. It commences with violent and repeated ſhiverings, ſucceeded by fluſhes of heat; with pains of the head, neck, and limbs; roughneſs of the throat; a dark redneſs and enlargement of the papillæ of the tongue; likewiſe an enlargement of the veins of the uvula, tonſils, &c. The formation of aphthæ is immediately followed by a dryneſs of the tongue, clammineſs of the mouth, nauſea, hiccough, heat in the ſtomach, which is increaſed by medicines, wine, or food, taken warm. A Diarrhœa ſupervenes, in which the ſtools are of a dark brown colour, and often ſtreaked with blood. The urine is at firſt clear, but has afterwards a curdly pink ſediment, as in other hectic caſes. There is uſually pain and deafneſs in one ear, with great pain and tenderneſs in the ſoles of the feet. A circumſcribed redneſs appears on the cheeks towards evening, attended with a quick pulſe, heat of the ſkin, ſlight delirium, and reſtleſſneſs. During the day the patient is languid and heavy, and ſometimes thirſty, with but little appetite. After the tongue, fauces, &c. have been healed, the Aphthous ulcerations return again, with internal heat, general uneaſineſs, and the ſame train of ſymptoms as at firſt. By frequent relapſes of this kind, the patient is often reduced to an extreme extreme degree of debility and emaciation; and the whole duration of the complaint is from five to twelve weeks. The cafes of Hectic, put down in the laft Report for the year 1800, were moftly of the kind here defcribed. An account of this complaint was, by miftake, omitted in its proper place.

"In the lifts of difeafes prefixed to the Reports, two-thirds of the cafes occurred among the lower claffes of people, moftly patients of the Public Difpenfary, near Temple-Bar: the remaining third was the refult of private practice in the upper ranks of fociety. The increafed number of cafes put down for the laft two years muft be in a great meafure referred to the flourifhing ftate of the Difpenfary, by means of which the medical attendants became more fully acquainted with the difeafes of the diftrict; a diftrict extending from Smithfield and St. Paul's to St. Martin's Lane and Tottenham-Court Road.

"A comparative view of the Bills of Mortality, at different periods, and a diary of the weather, are given at the end of the book, as neceffary appendages to a work of this kind. The Meteorological Journal, taken from the Philofophical Tranfactions, was kept at the apartments of the Royal Society. It is to be noticed, that the quickfilver in the bafon of the barometer is 81 feet above the level of low water fpring-tides at Somerfet-Houfe."

The obfervations interfperfed through the work are every where judicious and important, and the neceffary tables and indices are not omitted.

---

*An Introduction to a Course of Lectures on the Operations of Surgery*; by T. Chevalier, A. M. &c. 8vo. pp. 58. London, 1801, Bagfter, Callow, &c.

We advife every young man who wifhes to become a refpectable furgeon, to read this lecture more than once. It embraces a concife view of the following fubjects, viz. The natural endowments, acquired preparatory knowledge, neceffity of anatomical knowledge, phyfiology, appearances of difeafe, juft reafoning, remedies, operations on the dead and living body. On this part of the duty of a good furgeon, Mr. C. points out a number of confiderations refpecting the circumftances of the patient; the inftruments, unforefeen accidents during an operation, general rules for conducting an operation, the after treatment, and fupervening fever. Mr. C. concludes with inculcating the neceffity of induftry and diligence, from the commencement of his ftudies to the end of a furgeon's profeffional labours. As a fpecimen of Mr. C's ftile and manner, we fhall prefent our readers with his obfervations on what regards the patient who is to undergo an operation.

"Having faid fo much on thofe things which fhould be attended to by a furgeon refpecting his own qualifications, let us now confider what is neceffary for him to regard refpecting his patient.

"Before undertaking any important operation feveral things are to to be confidered. Thefe have been briefly expreffed in the following Latin verfe, which our forefathers have handed down to us:

*Quis, quid, ubi, quibus auxiliis, cur, quomodo, quando.*

"The ftate or condition of the patient is a matter of great importance, efpecially in thofe operations which are rendered neceffary by previous difeafe, and are not fuddenly required by ferious accidents. It is proper efpecially to attend to his age, conftitution, and to the prefent ftate of his general health; whether he be ftrong or debilitated, irritable and low; whether he appear fully equal to the operation, or whether there be any probability of his dying fooner in confequence of it.

"With regard to the difeafe itfelf, not only its nature is to be taken into the account, but alfo its particular fituation; the length of time it has continued, and whether it be fimple or accompanied with any peculiar circumftances which render a difference in the operation, or in the treatment, neceffary.

"Why the operation fhould be performed is another confideration. Is it abfolutely neceffary? Is there any rifk incurred by it? And if there be, Is the advantage expected fully equal to that rifk? and is the probability of fuccefs fufficient to juftify the expofure to it?

"In what is the operation itfelf to confift? and in what manner may it be moft advantageoufly executed? By what inftruments? With what dreffing and bandage? Is any preparation by medicine needful? What attendants will be required to do all that is to be done, without confufion and embarraffment?

"What fituation is moft proper? On a bed, a table, or a chair? How fhould the patient and the operator be placed with refpect to each other, and with refpect to the light? And to what fituation fhould the patient be removed afterward, and that he may be kept quiet, and under circumftances the moft favourable to fuccefs?

"When fhould the operation be done? Is it neceffary immediately, or how long may it be fafely delayed; and what advantages are to be gained by delaying it, or what by performing it early?

"Let it not be thought that by confidering thefe queftions, an operator muft become hefitating, timid, or unfettled. By no means. This can only happen to weaker minds. He who is habituated to reflect on thefe points with care, will not be on that account lefs prompt, bold, or firm, where promptitude, boldnefs, and firmnefs are required. The faculty of confidering and judging, though complex and numerous in its operations, is comparatively eafy when fully attained. Its different fteps pafs through the mind with wonderful rapidity. Like a new language, it confifts of many parts, many words, and many idioms, which muft firft be learned by much application, pains, and practice; but when it is once acquired, the ufe of it is as eafy as it is agreeable and ufeful.

"Suppofing then an operation to be clearly indicated, and the period proper for performing it fufficiently evident, the next thing neceſſary is to prepare the mind of the patient, and to communicate to him the opinion that is formed on his caſe, with ſufficient delicacy, and yet with firmneſs. Let it be conſidered that the patient is an human being, poſſeſſed of the faculty of reaſon, alive to the ſenſe of pain, who has the greateſt burden of the caſe to bear, and demands all that tenderneſs from the ſurgeon which the ſurgeon would himſelf deſire in like circumſtances. If therefore the patient ſhould chance to be heſitating and irreſolute, if at the ſame time he ſhould be intelligent and judicious, the nature of the caſe may as far as poſſible be explained to him, and he may be reaſoned with reſpecting the neceſſity and urgency of the operation. But in reaſoning with a patient on ſuch a ſubject, the ſurgeon, who before has weighed all the circumſtances of the caſe, muſt not waver to the caprices of his patient, nor appear as though he were in doubt, merely becauſe his patient is ſo. Having once clearly, and on mature judgment, ſtated his opinion, unleſs any thing turn up really to demand a reconſideration of it, he will only diminiſh the reſpect and confidence which ought to be repoſed in him, by appearing over candid and pliable.

"But as all human judgment is liable to error, it is ſeldom proper, eſpecially in dangerous or complicated caſes, to refuſe a conſultation with any perſon of fair character and approved abilities. In moſt caſes, before any of the more ſerious operations, particularly when the patient is a perſon of rank, or of large connections in life, two opinions are more ſatisfactory than one. The blame which illiberal and uninformed people are always ready to throw on the want of ſucceſs, and the ſuſpicions which may ſometimes ariſe in the minds of perſons of a more honourable character, who may hear the caſe but imperfectly detailed, and yet be aſked for their opinion, will be more effectually precluded by the concurrence of two perſons, than by the ſingle judgment of one; eſpecially if he be young, comparatively of but little experience, or but little known.

"In peruſing the works of ſome of the older ſurgeons, one is almoſt frightened to ſee the load of inſtruments they have delineated; and can ſcarcely help ſighing over the miſerable patients who were ſo unfortunate as to be ſcrewed, and bored, and mangled, with what look more like machines for torture than inſtruments of relief. No doubt, in many of their contrivances, they totally loſt ſight of the difference between a dead and a living body, and reaſoned like carpenters, rather than ſurgeons. They were alſo too fond of parade, and a pompous appearance. If a knife were not crooked, and its handle carved, and its caſe of a certain formal make, they would almoſt have diſdained to uſe it. But "the more ſimple our inſtruments are, the better. "Our fingers are our firſt and principal inſtruments; and that "capacity and knowledge by which they are to be guided, "is the univerſal inſtrument, without which all the reſt will "be uſeleſs."

"Sometimes during the performance of an operation, accidents occur, occur, which greatly try the firmneſs, patience, and ability of the ſurgeon. The diviſion of a large artery unexpectedly, or the diſcovery of new circumſtances in the diſeaſe, tend naturally to confuſe and agitate the mind. But at the moment when theſe things occur, he ſhould endeavour to recollect that time is not now to be waſted by regretting what is paſt, or thinking how it might have been avoided—All that will be proper afterward; but now, he muſt promptly and at once conſider what is actually the caſe, juſt as if he were then firſt called to it, and act according to the preſent exigency. In this power of ſuddenly turning the mind off from the former appearance of the caſe to the preſent, juſt as it would be turned to the caſe of another patient who might preſent himſelf, conſiſts much of that ſkill and ſelf-poſſeſſion, which has uſually diſtinguiſhed great operators. It is ſaid of that moſt accompliſhed admiral, Sir Francis Drake, who experienced the greateſt reverſes and difficulties in his profeſſion, perhaps, of any man on record, that when thrown into a new and perplexed ſituation, his mind immediately, and almoſt inſtinctively, went to work, contriving how to extricate himſelf from it; and that he was never heard on ſuch occaſions to ſpend a moment in reflecting on what was paſt, or ſpeculating on what might come, but was at once, inſtantaneouſly, as it were, and wholly employed in thinking what was to be done under the exiſting circumſtances, juſt as though he had expected them to happen. Such a diſpoſition I would recommend every ſurgeon to cultivate; but if he would ſucceed in this attempt, he muſt remember, it can only reſult from a thorough knowledge of his profeſſion, and is not to be the reward of ignorance, or raſhneſs."

